# Should we delay leaf water potential measurements after excision? Dehydration or equilibration?

**DOI:** 10.1186/s12870-024-05756-4

**Published:** 2024-11-08

**Authors:** Alicia V. Perera-Castro, Jaime Puértolas, Beatriz Fernández-Marín, Águeda M. González-Rodríguez

**Affiliations:** 1 Department of Botany, Ecology and Plant Physiology, Universidad de La Laguna (ULL), La Laguna, Canary Islands, 38200 Spain; 2 Institute of Biology, University of Hohenheim, Stuttgart, Germany; 3 Department of Plant Biology and Ecology, University of the Basque Country (UPV/EHU), Leioa, Bilbao, 48940 Spain

**Keywords:** Leaf storage, Leaf water potential equilibration, Leaf water content, Pressure chamber, Transpiration, Vaseline

## Abstract

**Background:**

Accurate leaf water potential (Ψ_w_) determination is crucial in studying plant responses to water deficit. After excision, water potential decreases, even under low evaporative demand conditions, which has been recently attributed to the equilibration of pre-excision Ψ_w_ gradients across the leaf. We assessed the influence of potential re-equilibration on water potential determination by monitoring leaf Ψ_w_ and relative water content decline after excision using different storage methods.

**Results:**

Even though leaf Ψ_w_ declined during storage under low evaporative demand conditions, this was strongly reduced when covering the leaf with a hydrophobic layer (vaseline) and explained by changes in relative water content. However, residual water loss was variable between species, possibly related to morpho-physiological leaf traits. Provided water loss was minimized during storage, pre-excision leaf transpiration rate did not affect to the magnitude of leaf Ψ_w_ decline after excision, confirming that transpiration-driven Ψ_w_ gradients have no effect on leaf Ψ_w_ determination.

**Conclusions:**

Disequilibrium in water potentials across a transpiring leaf upon excision is dissipated very quickly, well within the elapsed time between excision and pressurization, therefore, not resulting in overestimation of leaf Ψ_w_ measured immediately after excision. When leaf storage is required, the effectiveness of a storage under low evaporative demand varied among species. Covering with a hydrophobic layer is an acceptable alternative.

**Supplementary Information:**

The online version contains supplementary material available at 10.1186/s12870-024-05756-4.

## Background

Measuring plant water potential (Ψ_w_) is essential to understand plant responses to the environment. Water potential determines many physiological responses in the plant, making measurement accuracy crucial ever since the development of the main measuring methods such as the psychrometer and the pressure chamber [1–7]. Since most of the measuring procedures involve the excision of the organ prior to its measurement, water loss between excision and measurement can be considered as the main source of error in Ψ_w_ determination. This is particularly important for tissues with high transpiration rate such as leaves. Within the first 30 s after excision, Ψ_w_ can drop around 0.7 MPa in high transpiration plants depending on leaf elasticity [8, 9]. This prescribed the use of semi-portable measuring devices such as the pressure chamber, which has been transported to the field, even into remote areas, in ecophysiological studies. Nevertheless, several studies have demonstrated that preventing water loss by different techniques might be effective for accurate Ψ_w_ measurements [6, 8, 10, 11].

Some reports and theoretical studies indicate that Ψ_w_ gradients exist within a transpiring leaf [12–14], provoked by the resistances to flow of the apoplastic and symplastic pathways compiled between the xylem and the mesophyll cell walls. Empirical studies have though demonstrated that these gradients disappear within the order of seconds after excision [15, 16], which is generally shorter than the time taken for a pressure chamber [17] or psycrometer [18] measurement. Additionally, modelling of Ψ_w_ distribution in a transpiring leaf demonstrates the existence of large gradients of Ψ_w_ between xylem and epidermal cells, which are proportional to the magnitude of the transpiration flow [19]. Consistently, relative humidity in the intercellular air spaces inside leaves has been recently shown to decrease in high air vapour pressure deficit [20, 21]. As resistances within the midrib and near the edge of a leaf are large (≈ 0.8 MPa [14]), the equilibration of Ψ_w_ within the leaf can take more time than that required for equilibration between symplast and apoplast, as suggested by Turner [22, 23]. If that occurs, Ψ_w_ measurements with the pressure chamber technique would reflect that of the wettest cells and not the entire leaf [22]. Therefore, this can be termed as water potential disequilibrium, as the water potential is not equilibrated across the leaf and tend to equilibrate after excision when transpiration has ceased. Some observations that suggest overestimation of leaf Ψ_w_ when measured immediately after excision could be explained by this process [22, 24]. Therefore, while measuring immediately after excision would minimize water loss from leaf tissues, the potential existence of Ψ_w_ disequilibrium might prescribe delaying measurements to allow water redistribution within the leaf.

Even though the existence of internal disequilibrium in water potential and the time after excision required to achieve equilibration is an important methodological issue, it has been seldom addressed in the literature. Some studies suggested the potential existence of this phenomenon in the early reports of Ψ_w_ determination [22, 24–26]. More recently, a wide study on leaf Ψ_w_ procedures reported large differences in Ψ_w_ (0.31 ± 0.1 MPa) between leaves of *Olea europaea* measured immediately and 30 min after excision [6]. These changes in Ψ_w_ over the time were attributed to post-excision equilibration of the transpiration-driven Ψ_w_ gradients occurring before excision. To reach that conclusion, the authors assumed that the cool humid air inside a hermetically closed bag where leaves are stored provides very low vapour pressure deficits, so leaf water loss is negligible and therefore dehydration do not explain the observed Ψ_w_ decline. Putative leaf dehydration would also explain not only this decline but also its positive relationship with stomatal aperture at the time of excision reported also in *Olea europaea* [6]. Moreover, transpiring leaves of other species measured in the abovementioned study and in other earlier studies using slightly different or even similar storage techniques did not change appreciably their Ψ_w_ with time [10, 27, 28], which excludes the possibility of water potential overestimation due to disequilibria, at least within the normal operational time between excision and measurement. This suggests the possibility that dehydration during storage conditions can be the cause of water potential decline. However, since leaf dehydration has not been measured in these studies, the doubt about the cause of Ψ_w_ decline remains unsolved.

The aim of this study was to assess the existence and extent of overestimation of leaf Ψ_w_ due to immediate measurements of water potential after excision in different species, in comparison with delayed measurements. Concomitantly, we compared the effects of different leaf storage methods on leaf dehydration. We hypothesized that 1) when leaf dehydration is minimised during storage, Ψ_w_ decrease after excision is also reduced. Using the most effective procedure in reducing dehydration, changes in leaf Ψ_w_ and fresh weight were recorded immediately after excision, monitored during post-excision storage, and compared with those recorded in non-transpiring leaves to assess whether variation of Ψ_w_ was explained by dehydration or by internal re-equilibration. We hypothesized that any potential decrease in leaf Ψ_w_ after excision can be explained by concomitant decrease in relative water content, and this relationship is similar in transpiring leaves immediately after excision and in non-transpiring equilibrated leaves.

## Methods

### Experimental design

Three different experiments were performed. The first experiment aimed to compare the effectiveness of two methods of leaf storage in preventing leaf dehydration (Experiment 1). The two following experiments (Experiments 2 and 3) used the most effective method of storage according to the results of Experiment 1 to assess the putative effect of internal disequilibrium on leaf water potential determination in transpiring leaves.

For all experiments, well-watered ornamental plants growing in gardens at the University of La Laguna campus (Tenerife, Spain) were selected to represent a wide range of leaf mass per unit area, including tree, shrub, herbaceous and fern species (see species list at each experiment section). Leaf Ψ_w_ was periodically measured from 11:00 to 13:00 h by the same operator at different moments after excision (see details for each experiment) with a pressure chamber (model 1505D-EXP, PMS Instrument Company, Albany, USA) applying a stable rate of pressurization (0.01 MPa s^− 1^). A moist paper was placed inside pressure chamber to minimize dehydration during measurements (Fig. S1). At each measurement time (*t*), leaf fresh weight (FW_t_) was determined with a precision portable balance (ML203E, Mettler Toledo International Inc., Ohio, USA). Then, each leaf was sealed within a 50 mL-Falcon tube with the cut end of the petiole submerged in water to rehydrate for 8 h and weighed again to obtain its saturated weight (SW). After 48 h of being desiccated in an oven at 60 °C, dry weight (DW) was measured. Therefore, the relative water content at time of measurement (*t*) (RWC_t_) was calculated as [29]:

$$RW{C_t}\left( \% \right) = 100\cdot{{F{W_t} - DW} \over {SW - DW}}$$

### Experiment 1. Comparison between methods of leaf storage

The decrease in leaf Ψ_w_ and RWC during one hour of storage after excision was assessed in leaves from five plants of four different species (*Solanum nigrum* L, *Morella faya* (Aiton) Wilbur, *Prunus lusitanica* L and *Scrophularia glabrata* Aiton). Leaf storage was conducted using two different methods termed here “simple humid bag” method and “humid bag plus vaseline” method. Firstly, Ψ_w_ of one leaf per species and plant/branch was measured immediately after excision (after 2 min maximum, Ψ_w,0_). Within 4–5 min after the excision of the first leaf, two adjacent leaves per plant/branch were excised, weighed to calculate initial RWC (RWC_0_) and stored according to each of the two methods. The “simple humid bag” method is described by Rodríguez-Domínguez et al. [6]. Briefly, after weighing, the excised leaf was enclosed in an unsealed plastic bag wrapped in humid paper towel, which, in turn, was sealed into a zip-lock plastic bag and introduced into a cool box. In the “humid bag plus vaseline” method, after weighing, the leaf was covered with a vaseline layer and wrapped with aluminium foil (to avoid losses of vaseline mass during manipulation and storage), leaving the most proximal 1–1.5 cm of the petiole free from vaseline (to avoid lubricating the sealing gasket of the pressure chamber). After wrapping in foil, the leaf was weighed again to obtain the vaseline + aluminium weight and stored for one hour inside the double bag and the cool box as described above. The procedure was repeated five times, always alternating the storage method. Leaf Ψ_w_ and weight were measured again after 1 h of storage (Ψ_w,1 h_¸RWC_1h_). Dry weight was measured as described above, but vaseline was removed before drying by immersing and shaking the leaf three times inside clean hot water (70–90 °C) for 1 min at least. Variation of Ψ_w_ and RWC after 1 h were calculated as:

$$\Delta {\Psi _w} = {\Psi _{w,1h}} - {\Psi _{w,0}}$$

$$\Delta RWC = RW{C_{1h}} - RW{C_0}$$

Note that ΔΨ_w_ was calculated comparing values from two different adjacent leaves collected simultaneously, while ΔRWC compares values taken in the same leaf. Therefore, leaf water status variability within a plant is also included in ΔΨ_w_ variance. A scaled photograph of five intact leaves was taken to calculate the leaf area with ImageJ (NIH, LOCI, University of Wisconsin). Leaf mass per area (LMA) was calculated as the ratio of DW to leaf area.

Additionally, to assess the extent of the potential water loss through the uncovered petiole, the percentage of weight loss 1 h and 4 h after excision was monitored for leaves of *S. nigrum* and *P. lusitanica* stored with the vaseline method as described above and for leaves stored completely covered with vaseline, petiole included. In both cases, the weight of leaf, vaseline and aluminium foil was measured immediately after excision (*W*_0_) and afterwards. The relative water loss was calculated as:

$$\:Relative\:water\:loss\:\left(\%\right)=100\frac{{W}_{0}-{W}_{t}}{SW-DW}$$

Where *W*_*t*_ is the weight of leaf (excluding vaseline and aluminium foil) recorded at time *t* = 1–4 h after excision.

### Experiment 2. Monitoring water potential and water content with time

A more detailed monitoring of Ψ_w_ and RWC after excision was performed for *Viburnum rigidum* Vent, *Solanum nigrum* L and *Prunus lusitanica* L using the most effective storage method in preventing dehydration, i.e. “humid bag plus vaseline” method (see Results). This time, after weighing after excision as described above, Ψ_w_ of transpiring leaves was measured either immediately after excision (within 30 s approximately), or later, at 20 min, 50 min and 4 h after excision (*n* = 5 per species and time). Excision of leaves was organized so that the replicates of each time-group alternated each other in order to avoid possible time-dependent effect. As for Experiment 1, stored leaves were weighed again after each Ψ_w_ measurement. ΔΨ_w_ and ΔRWC were calculated for each moment as:

$$\Delta\:{\Psi\:}_{w,t}={\Psi\:}_{w,t}-{\Psi\:}_{w,0}$$

$${\Delta\:RWC}_{t}={RWC}_{t}-{RWC}_{0}$$

where *t* is the moment at which the second measurement of Ψ_w_ and RWC was taken: 20 min, 50 min–4 h after excision. Five (*S. nigrum* and *P. lusitanica*) and three (*V. rigidum*) additional transpiring leaves were collected and sealed inside 50 mL-Falcon tubes with the cut end of the petiole submerged in water for 8 h. Then, pressure-volume measurements were performed following the bench dehydration method [30] covering the range of Ψ_w_ values observed in Experiment 2.

### Experiment 3. Determining the effect of initial transpiration on water potential variation during storage

Leaf water potential variation during storage was assessed in transpiring and non-transpiring leaves of five distant branches (*n* = 5) of one plant of *Cheirolophus canariensis* (Brouss. ex Willd.) Holub and *Hibiscus rosa-sinensis* L, and five plants of the fern *Davallia canariensis* (L.) Sm. In one leaf of three of the plants/branches per species (*n* = 3), transpiration was suppressed by wrapping leaves in aluminium foil for at least 30 min. In another adjacent leaf per plant/branch, transpiration rate (*E*) and stomatal conductance (*g*_s_) were measured with an open gas exchange system (Li-6800; Li-Cor Inc., USA). Air humidity, temperature and light radiation were set automatically by the system to meet external conditions outside the chamber (25–30 °C, 45–63% of relative humidity). CO_2_ concentration inside the chamber was set to 400 µmol mol^− 1^ and flow to 400 µmol s^− 1^. After the dark period, wrapped leaves were unwrapped and immediately inserted in the gas exchange cuvette and measured under the same conditions, except for light radiation, which was set to zero. After gas exchange measurements, Ψ_w_ was measured either immediately after excision or after 1 h of storage in one transpiring and another adjacent non-transpiring leaf per plant/branch. Leaves were stored according to the “humid bag plus vaseline” method.

### Statistical analysis

For each species and method, differences between Ψ_w,0_ and Ψ_w, t_ (ΔΨ_w_) of the same plant/branch and between RWC_0_ and RWC_t_ (ΔRWC) of the same leaf observed in the three experiments were tested by determining the difference between deltas and zero with a one-sample t-test. To test the effect of the storage method on Ψ_w_ and RWC a two-way repeated measures ANOVA considering branch/plant as the experimental unit were conducted. For all experiments, measurement time was considered a within-subject factor. Storage method in experiments 1 and 2, transpiration status in experiment 3 were also considered within-subject factors to test the effect of storage or transpiration on the variation of Ψ_w_. A non-linear regression between RWC and Ψ_w_ from the P-V curves was performed fitting quadratic functions to the data. All analyses were performed using the R statistical software (1.4.1717 version) [31], using either in-built packages or *rstatix* [32].

## Results

### Experiment 1. Storage in a sealed bag with humid air is not always enough for avoiding dehydration of samples

**Fig. 1:**
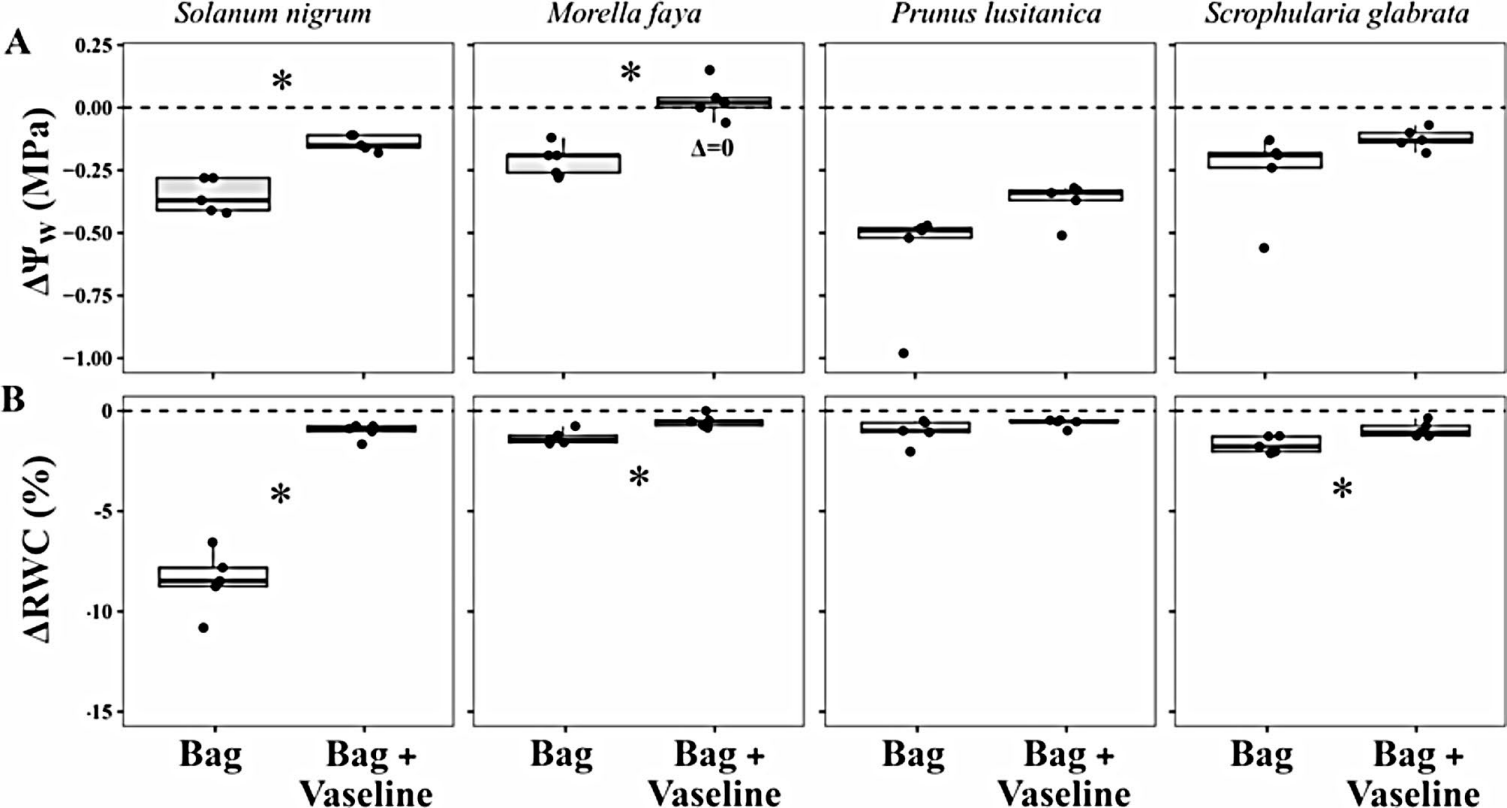
Decrease in leaf water potential (ΔΨ_w_, **A**) and relative water content (ΔRWC, **B**) between the initial value measured immediately after excision and after one hour of storage under either the “simple humid bag” method (Bag) or the “vaseline plus humid bag” (Bag + Vaseline) method of leaf storage (*n* = 5). Asterisks indicate significant interactions between the two within-subject factors (storage method and measurement time) in the two-way repeated measures ANOVA (*P* < 0.05). The symbol Δ = 0 denotes non-significant differences between zero and the average value of ΔΨ_w_ (one-sample t-test; *P* > 0.05). All average ΔRWC values were statistically different from zero. Note that ΔΨ_w_ was calculated comparing values from two different adjacent leaves collected simultaneously, while ΔRWC compares values taken in the same leaf

For both methods of storage, all studied species decreased significatively Ψ_w_ after one hour of storage (a decrease between 0.12 and 0.59 MPa, *P* < 0.01, one-sample t-test), except for *M. faya* leaves stored with the “humid bag plus vaseline” method (Table S1, S2). However, covering with vaseline generally resulted in a smaller decrease in leaf Ψ_w_ compared to the simple bag method, with significant differences between methods of storage in two of the four species: *S. nigrum* and *M. faya* (Fig. 1A, Table S3).

In accordance with Ψ_w_, ΔRWC was significantly minimised when leaves were stored with vaseline in three of the studied species (all except *P. lusitanica;* Fig. 1B). It is noteworthy to consider here that *S. nigrum*, *M. faya* and *S. glabrata* presented values of LMA of 23.5 ± 3.9, 96.5 ± 1.13 and 109.9 ± 12.4 g·m^− 2^, respectively, while LMA of *P. lusitanica* was remarkably higher: 152.5 ± 6.7 g·m^− 2^. Average ΔRWC of leaves stored with vaseline was significantly differing from zero in all cases (*P* < 0.05), and were ≤ 1% for all species (1.01, 0.51, 0.59 and 0.93% for *S. nigrum*, *M. faya*, *P. lusitanica* and *S. glabrata*, from an initial averaged RWC of 96.7, 89.4, 97.6 and 89.8%, respectively). It must be noted that ΔRWC was calculated from values measured on the same leaf, meanwhile ΔΨ_w_ was derived from values of different but adjacent leaves. Therefore, both leaf-to-leaf variability and measurement error contribute to the actual difference between initial and final Ψ_w_ value, while only measurement error contributes to RWC.

**Fig. 2:**
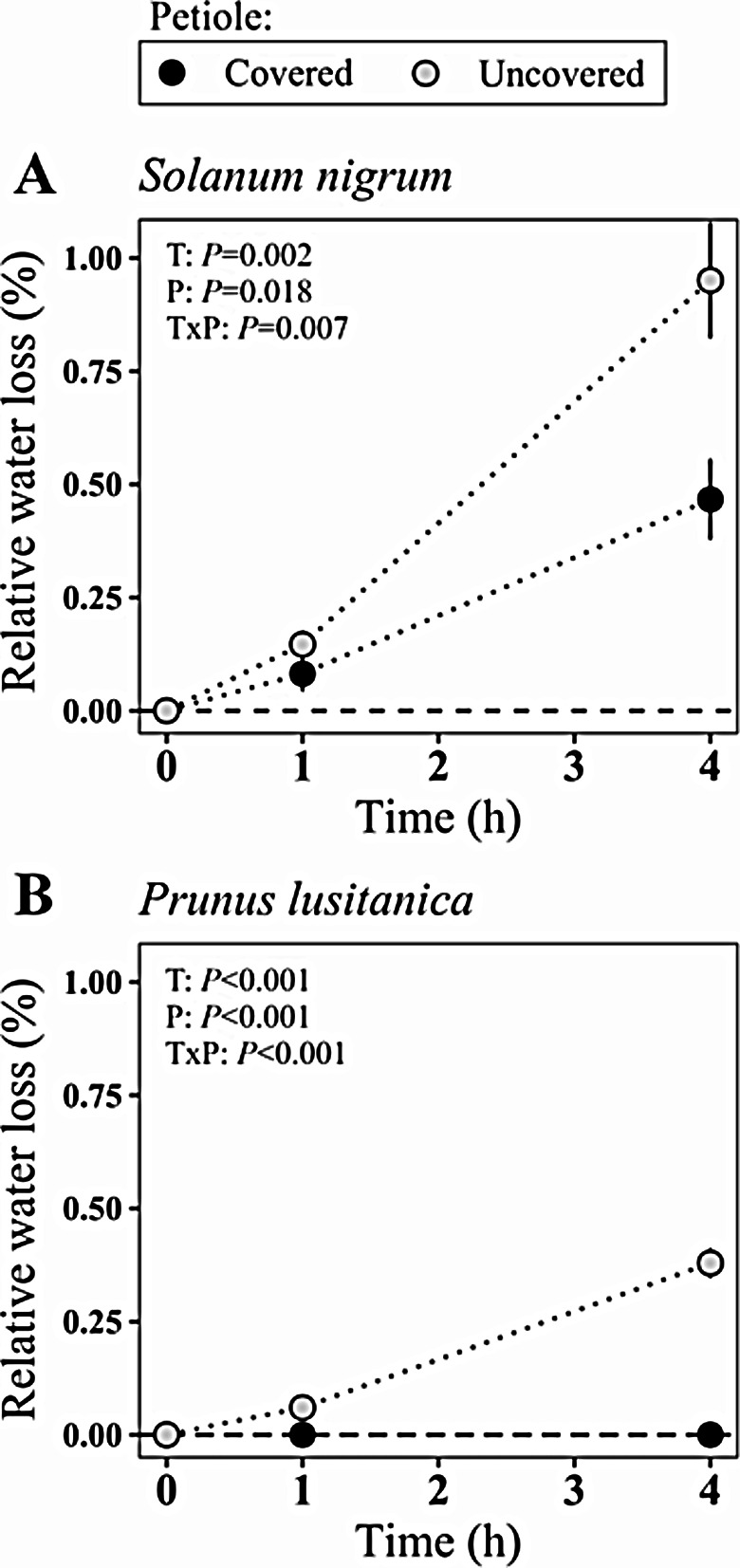
Variation (mean ± s.e., *n* = 5) in relative water loss of leaves stored with the “vaseline plus humid bag” method of (**A**) *Solanum nigrum* and (**B**) *Prunus lusitanica* after excision. During storage, petiole of leaves was (black dots) or was not (grey dots) covered in vaseline. Results of two-way repeated measured ANOVA model are shown in the upper right corner of each panel

Vaseline applied to petioles significantly reduced relative water loss in the two studied species: *S. nigrum* (Fig. 2A) and *P. lusitanica* (Fig. 2B). Moreover, the weight of petiole-covered leaves of *P. lusitanica* did not significantly change 4 h after excision (*P* = 0.978) (Fig. 2A). However, even vaseline application to petiole could not completely prevent water loss in *S. nigrum*, (*P* < 0.001; Fig. 2B), although it was very small (0.4% of total leaf water weight).

### Experiment 2. Early water potential did not decrease when water loss was suppressed

**Fig. 3:**
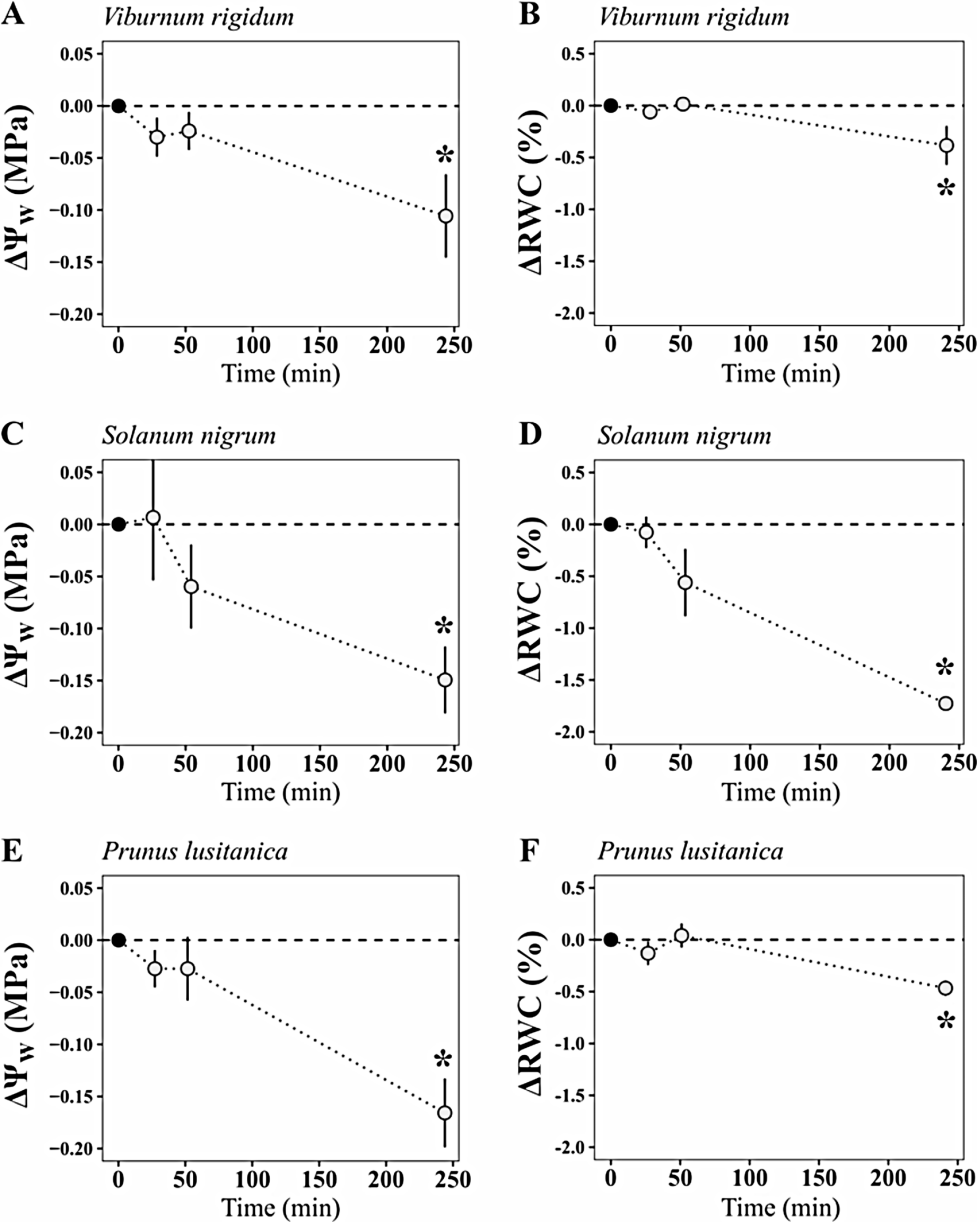
Evolution (mean ± s.e., *n* = 5) of the difference in water potential (ΔΨ_w_; **A**, **C**, **E**) and relative water content (ΔRWC, **B**, **D**, **F**) at 25, 50–240 min after excision of leaves stored with the “vaseline plus humid bag” method in *Viburnum rigidum* (**A**, **B**), *Solanum nigrum* (**C**, **D**) and *Prunus lusitanica* (**E**, **F**). Asterisks indicate significant differences between ΔΨ_w_ or ΔRWC and zero (one-sample *t*-test; *P* < 0.001). Note that ΔΨ_w_ was calculated comparing values from two different adjacent leaves collected simultaneously, while ΔRWC compares values taken in the same leaf

**Fig. 4:**
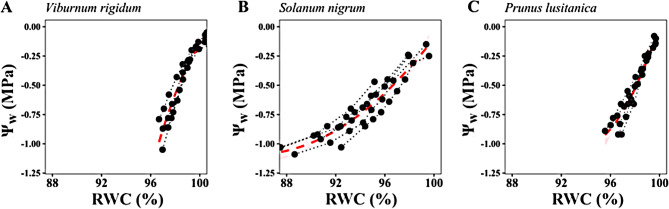
Relationship between leaf water potential (Ψ_w_) and relative water content (RWC) measured during progressive air-dehydration of leaves (*n* = 3–5) of (**A**) *Viburnum rigidum*, (**B**) *Solanum nigrum* and (**C**) *Prunus lusitanica* that were rehydrated for 8 h in 50 mL-Falcon tubes. Grey dashed lines represent quadratic fittings of Ψ_w_ with their respective 95% confidence intervals (pink-shaded areas): (**A**) y=-0.0303 × ^2^ + 6.21x-318.3 (*R*^2^ = 0.913), (**B**) y = 0.0046 × ^2^-0.788x + 32.5 (*R*^2^ = 0.897) and (**C**) y = 0.024 × ^2^-4.55x + 212.05 (*R*^2^ = 0.936)

In all tested species, significant (but small) variation of Ψ_w_ and RWC was observed only 4 h after excision (ΔΨ_4h_/ΔRWC_4h_ = 0.10 MPa/0.38%, 0.15 MPa/1.72% and 0.16 MPa/0.46% for *V. rigidum* -Fig. 3A, B-, *S. nigrum* -Fig. 3C, D- and *P. lusitanica* -Fig. 3E, F-, respectively). Changes between Ψ_w,0_ and Ψ_w,20 min,_ and between RWC_0_ and RWC_20min_ were not significant (ΔΨ_20m_ < 0.03 MPa; *P* > 0.184, ΔRWC_20min_ < 0.13%; *P* > 0.311 for the three studied species; Fig. 3). Such slight variations in Ψ_w_ and RWC were consistent with the relationship between RWC and Ψ_w_ observed in independent leaves for the three studied species (Fig. 4). According to those relationships, the observed ΔRWC_4h_ after storage (0.4–1.7% across species) predicted ΔΨ_w,4 h_ between 0.1 and 0.15 MPa, similar to the actual ΔΨ_w,4 h_ values.

### Experiment 3. Transpiring leaves did not differ from non-transpiring ones in their null variation of water potential

**Fig. 5:**
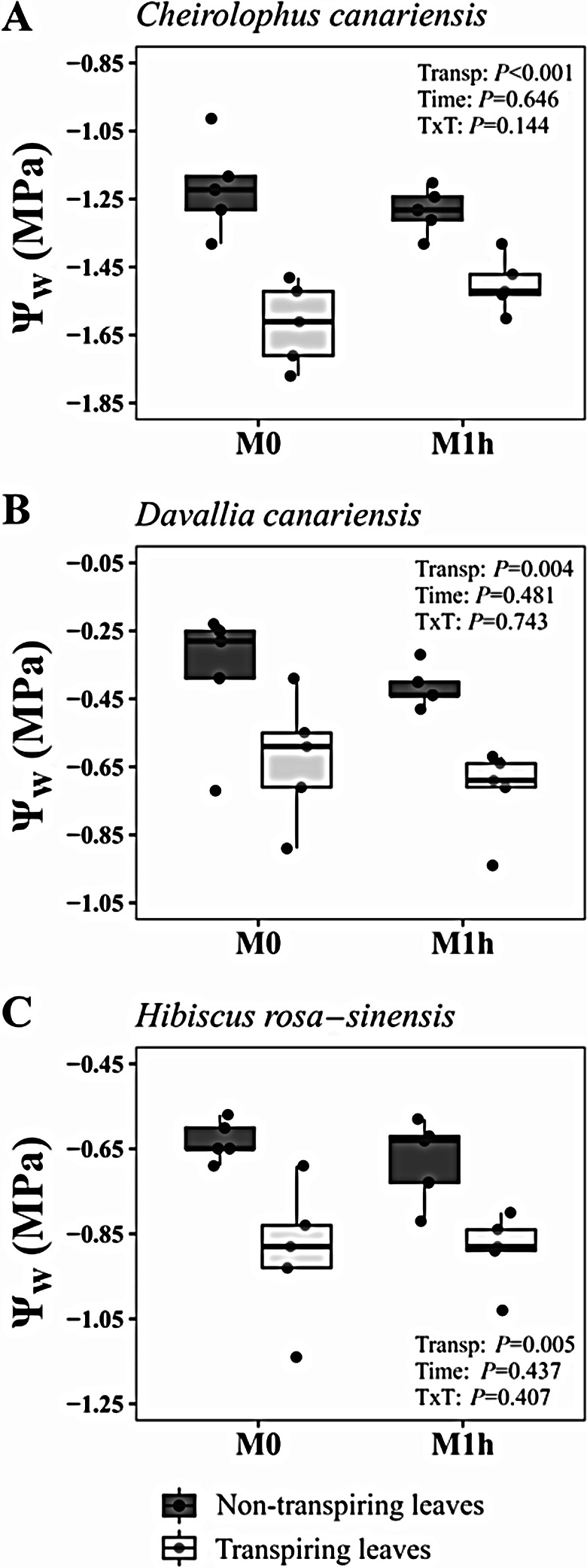
Water potential (Ψ_w_) of transpiring and non-transpiring leaves of (**A**) *Cheirolophus canariensis*, (**B**) *Davallia canariensis*, and (**C**) *Hibiscus rosa-sinensis* measured immediately after excision (M0) or in an adjacent leaf collected simultaneously but measured after 1 h of storage (M1h) following the “vaseline plus humid bag” method (*n* = 5). Non-transpiring leaves were covered with aluminium foil during 30 min before excision. Results of the two-way repeated-measures ANOVA models are shown in each panel

*C. canariensis*, *D. canariensis* and *H. rosa-sinensis* showed a significant suppression of transpiration and stomatal conductance after 30 min of dark adaptation (Table 1). As expected, Ψ_w_ was significantly higher in non-transpiring leaves than in transpiring ones, with an averaged difference of 0.48, 0.25 and 0.26 MPa in *C. canariensis*, *D. canariensis* and *H. rosa-sinensis*, respectively, for Ψ_w_ values measured 1 h after excision (Fig. 5). However, this difference in transpiration did not result in a significant effect on the interaction between transpiration status and time after excision in any of the studied species, being the variation of Ψ_w_ 4 h after excision null in both types of leaf (Fig. 5).

**Table 1:** Transpiration rate and stomatal conductance of transpiring and non-transpiring leaves

Species	Leaf treatment	E (mmol m^− 2^ s^− 1^)	g_s_ (mmol CO_2_ m^− 2^ s^− 1^)
*Cheirolophus canariensis*	Transpiring	7.9 ± 1.1*	340.9 ± 18.8*
	Non-transpiring	4.8 ± 1.1	164.0 ± 38.0
*Davallia canariensis*	Transpiring	1.7 ± 0.3*	42.8 ± 8.6*
	Non-transpiring	0.58 ± 0.04	15.1 ± 1.6
*Hibiscus rosa-sinensis*	Transpiring	0.96 ± 0.19*	39.0 ± 8.1*
	Non-transpiring	0.22 ± 0.01	10.9 ± 1.1

*Significant differences between transpiring and non-transpiring leaves (P<0.001, n=3)

## Discussion

### Changes in water potential: dehydration vs. equilibrium

This study found that putative re-equilibration of the leaf water potential gradient immediately after excision either has a negligible effect on determination of leaf water potential or it occurs within the seconds between excision and water potential determination. Water potential generally decreased with time after *t*_0_ (our first simultaneous leaf weight/water potential value), but this was always paralleled by reduced RWC (Fig. 1) and was negligible for the first 60 min (< 0.06 MPa of difference) in leaves stored with a method preventing dehydration for three-studied species (Fig. 3). Moreover, predictions of Ψ_w_ decrease with RWC [33] confirmed that the declining Ψ_w_ after excision could solely be explained by leaf dehydration (Figs. 3 and 4), which indicates that, at least in the studied species, the contribution of water potential equilibration across the leaf to the observed decrease in Ψ_w_ during storage is negligible. This suggests that disequilibrium of Ψ_w_ across the leaf upon excision, if existent, might relax very quickly, well within the elapsed time between excision and pressurization.

Our results (Fig. 1A) agree with recent reports in *Olea europaea* claiming that storage in a cool high humidity enclosure (similar to our “simple humid bag” system) did not prevent leaf water potential decline (between 0.2 and 0.5 MPa; [6]). This was attributed by the authors of the latter study to equilibration of water potentials across the leaf during the first 30 min after excision. Even though this or similar storage methods have proven effective to maintain stable water potential after excision in some studies [27, 28], some practical issues can induce substantial dehydration in leaves stored in humidified bags. For instance, Karlic and Hinckley [10] reports water transfer through plastic bags, which can be prevented using aluminium foil bags. It is also plausible that at the beginning of the storage period the air could not completely saturated as the bag needs to be transiently open, especially if sampling is carried out in a hot dry environment. In leaves of most angiosperms, excision even induces transient hydropassive stomatal aperture known as the “wrong-way” stomatal response or the Iwanoff effect [34–37]. All this might quickly dehydrate the leaf in the first minutes after excision. Therefore, any test of the potential effect of disequilibrium on leaf water potential determination needs to assess the extent of leaf water losses after excision and ideally use a storage method that minimises those losses. We demonstrate in this study that when using a storage method that prevents or minimises water losses for the first 60 min (even more effective when petiole is fully covered), Ψ_w_ remains stable after excision consistently across species. This agrees with early reports suggesting that Ψ_w_ can be stable after excision, provided appropriate methods of leaf storage that prevents its dehydration are followed [10]. In summary, recent reporting of water potential decrease in leaves stored in humid bags [6] has likely been misattributed to water potential equilibration across the leaf during storage, whereas, according to our study, it is probably caused by leaf dehydration.

### Storage with vaseline in transpiring leaves

Since the theoretical support for the existence of disequilibrium is based on the creation of water potential gradients due to effect of the transpiration flow across the leaf [19], leaf transpiration rate might be critical to determine the existence or not of disequilibrium. Thus, a recent study on the extent of this disequilibria in *Olea europaea* found no drop in Ψ_w_ during storage in humid bag when leaves were excised under low transpiration conditions, but a strong decrease when collection was carried out under high transpiration [6]. This transpiration-dependent decline of Ψ_w_ was attributed to water potential disequilibria at the time of excision under high transpiration and subsequent equilibration [6] during storage at null transpiration. Our study did not find any early decrease in Ψ_w_ during storage, either in transpiring or in non-transpiring leaves when using an effective method of water loss suppression (Fig. 5). While transpiring leaves of two of the studied species have low transpiration rates, sunlit *Cheirolophus* leaves exhibited high stomatal conductance and transpiration rates but still Ψ_w_ did not drop during storage. This indicates that the larger Ψ_w_ drop in transpiring compared to non-transpiring leaves stored in a humid bag without vaseline cover is solely related to higher leaf dehydration in the former. Thus, relaxation of water potential gradients between the xylem and the epidermal cells are likely to occur within seconds after excision and before the leaf is pressurised in the chamber.

### Possible reasons for the limitations of the “humid bag storage method”

The variation among species in their leaf water retention during storage ‒ highlighted in species with extremely different LMA and Ψ_w_-RWC relationship, such as *P. lusitanica* and *S. nigrum* ‒ may be related to differences in (1) their transpiration before excision, probably coordinated with leaf structure and cell water relations traits (especially leaf water content, capacitance and elasticity) as described for net CO_2_ assimilation [38–40], (2) stomatal response to light, and/or (3) leaf minimum conductance. Thanks to photoreceptors for blue and red light, stomata can usually respond rapidly to darkness and present a 70–100% of stomatal closure within 30 min, although there are ABA-mediated variation in the response time and even insensitive species [41–43]. A delayed response of stomata to darkness combined with a higher initial transpiration may lead to relevant losses of water in the first 20–30 min of storage. Furthermore, the closure of stomata is not always complete even when guard cells are fully deflated at zero turgor [44, 45]. Minimum leaf conductance is largely variable among plants [46, 47] and has been inversely associated with LMA [48], explaining the ineffectiveness of long-term storage for the low-LMA *S. nigrum* leaves even when fully covered with vaseline (Fig. 1). Knowing the sensitivity of the target species to storage is therefore essential to minimize potential errors if in situ measurements cannot be logistically possible, especially for species with low LMA.

### Final recommendations for water potential measurements

The implications of the results obtained here are especially important from a methodological point of view. The classic assumption that leaf water potential must be measured as soon as possible after excision [8] has been questioned recently [6]. According to the results of that study, not only adequate leaf storage conditions ensures that accurate leaf water potential measurements can be taken with the pressure chamber technique hours and even days after excision but also it is advised that measurements should not be taken before allowing enough time for equilibration of water potentials across different parts of the leaf. This substantially changes the experimental procedure for measurements not only under field conditions but also in the laboratory, and these recommendations are already being followed in plant physiology studies [49–51]. In contrast, our results demonstrate that even when storing the leaf under low VPD conditions, delaying measurements after excision might result in significant underestimation of Ψ_w_ (between 0.25 and 0.5 MPa in 1 h, Fig. 1). Nevertheless, we do not negate the possibility of storing leaves for posterior determinations of water potential, as some protocols have been proven effective in the past [10, 27, 28, 52]. However, we propose testing the suitability of the storage method by simply measuring the change in leaf relative water content during storage rather than assessing variations in water potential. This recommendation might be even more important for low LMA herbaceous species (such as *S. nigrum* in our study, see Fig. 2) if accurate Ψ_w_ determination is needed. Even though we have tested a range of different species collected at different transpiration status, the putative effect of re-equilibration on water potential measurement cannot be universally excluded. We also propose that investigation on that potential effect is conducted by first determining the most effective storage method as suggested above and then observing whether there are changes in water potential during storage.

In our study we found that, in addition to enclosure in a humid bag, storing the leaf covered with vaseline to effectively stabilise RWC and hence Ψ_w_ after excision. Covering the leaf with a vaseline layer might not only effectively prevent leaf water loss. The hydrophobic layer can also prevent the absorption of humidity during storage, which is common in many species with significant capacity for foliar water uptake [53, 54]. This method has proved to be very effective in sclerophyllous species, but in deciduous species showed to slightly increase water potential during storage [10]. Other methods like that proposed in the latter study of inserting the leaf in a sealed aluminium bag between wet mats or covering the leaf in paraffin [55] can also be effective, but as mentioned above, water loss should be tested prior to being used.

## Conclusions

In conclusion, disequilibrium in water potentials across the leaf upon excision, if present, did not result in overestimation of leaf Ψ_w_ for a range of species tested. Therefore, in contrast with recent suggestions to delay water potential measurements to allow for Ψ_w_ equilibration, we confirm earlier results recommending that leaf water potential measurements must be taken as soon as possible after excision. Whenever operational conditions do not allow measuring immediately after excision, we propose that, given the high variability in leaf water retention capacity, storage methods are tested appropriately, by assessing leaf water loss during the storage period rather than water potential changes. For further examination of putative disequilibria affecting water potential measurements, this procedure of storage assessment should be conducted before potential changes in Ψ_w_ during storage is determined.

## Electronic supplementary material

Below is the link to the electronic supplementary material.

Supplementary Material 1

## Data Availability

The datasets used and/or analysed during the current study are available from the corresponding author, Alicia V. Perera-Castro, on reasonable request, and supplementary Tables S1 and S4.

## Abbreviations

Ψ_w_: Leaf water potential
Ψ_w,0_: Leaf water potential measured immediately after excision
Ψ_w,t_: Leaf water potential measured after t time of storage
RWC: Relative water content
RWC_t_: Relative water content at t time of storage
ΔΨ_w_: Variation of leaf water potential during time of storage comparing values of two different adjacent leaves collected simultaneously
ΔRWC: Variation of relative water content of the same leave during storage
LMA: Leaf mass per area
