# Supplementary material for: Should we delay leaf water potential measurements after excision? Dehydration or equilibration?
Source: BMC Plant Biol. 2024 Nov 8;24:1056. doi: 10.1186/s12870-024-05756-4 (PMC11545772; doi:10.1186/s12870-024-05756-4)
Supplement: Supplementary file 1 — Supplementary Material 1 [file 12870_2024_5756_MOESM1_ESM.docx]

**Supplementary materials**

**Title: Should we delay leaf water potential measurements after excision? Dehydration or equilibration?**

**Authors**: Alicia V Perera-Castro, Jaime Puértolas, Beatriz Fernández-Marín, Águeda M González-Rodríguez


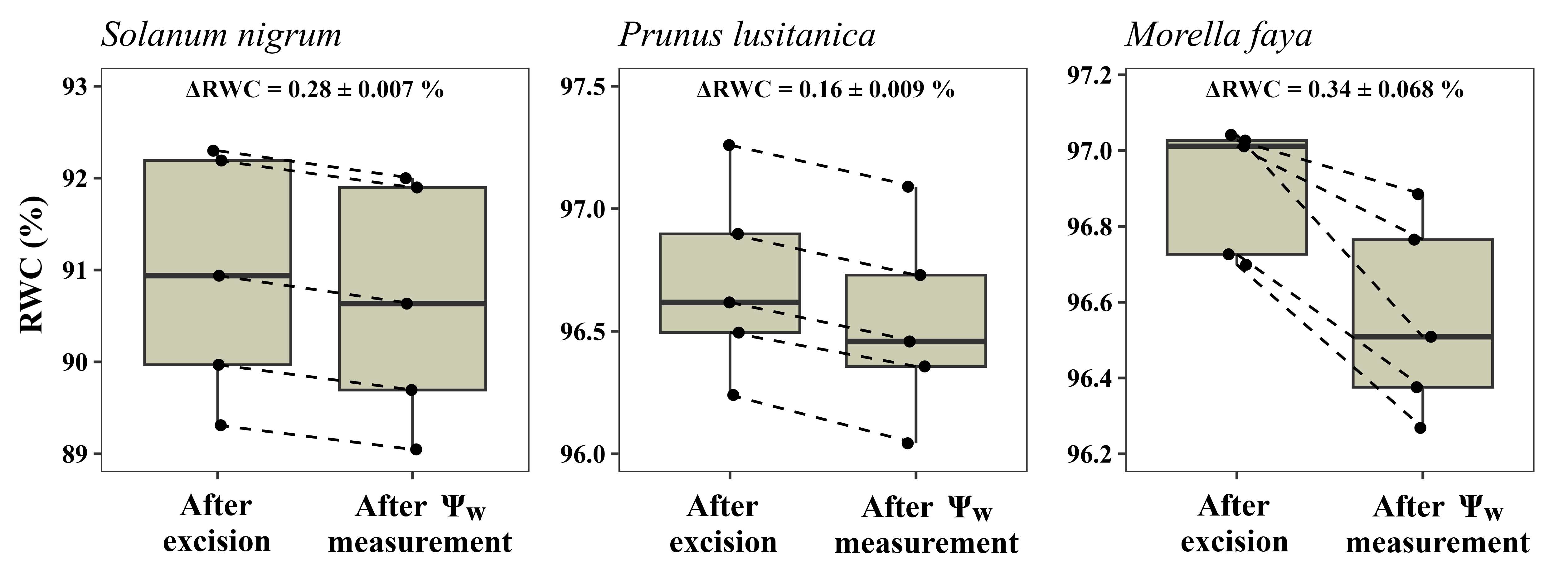


**Figure S1**. Variation of relative water content (RWC) of leaves as a consequence of water potential measurement in three studied species. Leaves were weighted immediately after excision and immediately after depressurization (2-3 minutes of difference). Water potential (-0.55 ± 0.02, -0.72 ± 0.02 and -0.46 ± 0.01 MPa, for *Solanum nigrum*, *Prunus lusitanica* and *Morella faya*, respectively) was measured by the same operator with a pressure chamber (model 1505D-EXP, PMS Instrument Company, Albany, USA) applying a stable rate of pressurization (0.01 MPa s^-1^). A moist paper was placed inside pressure chamber to minimize dehydration during measurements. The averaged drop of RWC (ΔRWC = RWC after excision – RWC after water potential determination) is shown in the upper side of each panel.

**Table S1.** Variation of water potential (Ψ_w_, MPa) and relative water content (RWC, %) after one hour of storage under either the “simple humid bag” method (Bag) or the “vaseline plus humid bag” (Bag+Vaseline) method of leaf storage. Note that ΔΨ_w_ was calculated comparing values from two different adjacent leaves (Ψ_w,0_ and Ψ_w,1h_) collected simultaneously, while ΔRWC compares values taken in the same leaf (RWC_0_ and RWC_1h_). ID corresponds to the branch were leaves of both treatments were collected.

| **Parameter** | **Storage** | **ID** | ***Solanum nigrum*** | | | ***Morella faya*** | | | ***Prunus lusitanica*** | | | ***Scrophularia glabrata*** | | |
| --- | --- | --- | --- | --- | --- | --- | --- | --- | --- | --- | --- | --- | --- | --- |
|  |  |  | **0** | **1h** | **Δ** | **0** | **1h** | **Δ** | **0** | **1h** | **Δ** | **0** | **1h** | **Δ** |
| Ψ_w_ | Bag | 1 | -0.59 | -0.87 | -0.28 | -0.91 | -1.19 | -0.28 | -0.42 | -1.4 | -0.98 | -0.7 | -1.26 | -0.56 |
|  |  | 2 | -0.65 | -1.02 | -0.37 | -0.89 | -1.08 | -0.19 | -0.42 | -0.89 | -0.47 | -0.85 | -0.98 | -0.13 |
|  |  | 3 | -0.61 | -1.03 | -0.42 | -0.96 | -1.15 | -0.19 | -0.65 | -1.14 | -0.49 | -0.76 | -0.95 | -0.19 |
|  |  | 4 | -0.56 | -0.97 | -0.41 | -1.06 | -1.18 | -0.12 | -0.43 | -0.95 | -0.52 | -0.78 | -0.96 | -0.18 |
|  |  | 5 | -0.64 | -0.92 | -0.28 | -0.82 | -1.08 | -0.26 | -0.41 | -0.89 | -0.48 | -0.85 | -1.09 | -0.24 |
|  |  | MEAN | **-0.61** | **-0.96** | **-0.35** | **-0.92** | **-1.13** | **-0.21** | **-0.46** | **-1.05** | **-0.59** | **-0.78** | **-1.04** | **-0.26** |
|  |  | SE | **0.01** | **0.03** | **0.03** | **0.03** | **0.02** | **0.02** | **0.04** | **0.09** | **0.09** | **0.05** | **0.05** | **0.07** |
|  | Bag+Vaseline | 1 | -0.47 | -0.58 | -0.11 | -0.93 | -0.89 | 0.04 | -0.41 | -0.78 | -0.37 | -0.79 | -0.89 | -0.1 |
|  |  | 2 | -0.60 | -0.76 | -0.16 | -0.84 | -0.82 | 0.02 | -0.53 | -0.85 | -0.32 | -0.77 | -0.84 | -0.07 |
|  |  | 3 | -0.59 | -0.74 | -0.15 | -0.80 | -0.65 | 0.15 | -0.39 | -0.72 | -0.33 | -0.75 | -0.89 | -0.14 |
|  |  | 4 | -0.61 | -0.72 | -0.11 | -0.82 | -0.82 | 0.00 | -0.41 | -0.75 | -0.34 | -0.8 | -0.93 | -0.13 |
|  |  | 5 | -0.59 | -0.77 | -0.18 | -0.71 | -0.77 | -0.06 | -0.38 | -0.89 | -0.51 | -0.7 | -0.88 | -0.18 |
|  |  | MEAN | **-0.57** | **-0.71** | **-0.14** | **-0.82** | **-0.79** | **0.03** | **-0.42** | **-0.79** | **-0.37** | **-0.76** | **-0.88** | **-0.12** |
|  |  | SE | **0.02** | **0.03** | **0.01** | **0.03** | **0.03** | **0.03** | **0.02** | **0.03** | **0.03** | **0.01** | **0.01** | **0.01** |
| RWC | Bag | 1 | 95.8 | 89.2 | 6.6 | 89.0 | 87.4 | 1.6 | 92.4 | 89.8 | 2.6 | 89.7 | 88.4 | 1.3 |
|  |  | 2 | 89.6 | 80.9 | 8.7 | 90.0 | 89.2 | 0.7 | 90.7 | 90.1 | 0.6 | 103.1 | 101.8 | 1.3 |
|  |  | 3 | 98.4 | 87.6 | 10.8 | 89.7 | 88.5 | 1.2 | 91.8 | 90.4 | 1.4 | 90.2 | 88.5 | 1.7 |
|  |  | 4 | 103.2 | 94.7 | 8.5 | 86.8 | 85.2 | 1.5 | 107.5 | 106.3 | 1.2 | 96.4 | 94.4 | 2.0 |
|  |  | 5 | 96.4 | 88.5 | 7.9 | 93.4 | 92.0 | 1.4 | 84.9 | 84.1 | 0.8 | 98.4 | 96.3 | 2.1 |
|  |  | MEAN | **96.6** | **88.1** | **8.5** | **89.7** | **88.4** | **1.4** | **93.4** | **92.1** | **1.3** | **95.5** | **93.8** | **1.7** |
|  |  | SE | **2.1** | **2.2** | **0.6** | **1.0** | **1.11** | **0.1** | **3.7** | **3.7** | **0.3** | **2.5** | **2.5** | **0.2** |
|  | Bag+Vaseline | 1 | 95.0 | 94.2 | 0.8 | 88.1 | 87.3 | 0.8 | 95.5 | 94.9 | 0.6 | 88.9 | 88.2 | 0.7 |
|  |  | 2 | 90.6 | 89 | 1.6 | 92.4 | 91.7 | 0.7 | 93.7 | 93.1 | 0.6 | 100.2 | 99 | 1.2 |
|  |  | 3 | 99.0 | 98.3 | 0.7 | 90.2 | 89.7 | 0.5 | 91.5 | 90.3 | 1.2 | 91.6 | 90.4 | 1.2 |
|  |  | 4 | 97.0 | 96.0 | 1.0 | 87.8 | 87.4 | 0.4 | 106.2 | 105.5 | 0.7 | 96.5 | 96.2 | 0.3 |
|  |  | 5 | 97.4 | 96.7 | 0.7 | 93.6 | 93.6 | 0.0 | 100.9 | 100.2 | 0.7 | 97.8 | 96.8 | 1.0 |
|  |  | MEAN | **95.8** | **94.8** | **1.0** | **90.4** | **89.9** | **0.5** | **97.5** | **96.8** | **0.7** | **95.0** | **94.1** | **0.9** |
|  |  | SE | **1.4** | **1.6** | **0.1** | **1.1** | **1.2** | **0.1** | **2.6** | **2.7** | **0.1** | **2.0** | **2.0** | **0.1** |

**Table S2.** Statistics results about the difference between ΔΨ_w_ and zero (one sample t-test, experiment 1).

| **Species** | **Storage** | **ΔΨ_w_** | **One-sample t-test** | |
| --- | --- | --- | --- | --- |
|  |  |  | **t** | **P** |
| *Solanum nigrum* | Bag | -0.35 ± 0.03 | -11.51 | <0.001 |
|  | Bag+Vaseline | -0.14 ± 0.01 | -10.19 | <0.001 |
| *Morella faya* | Bag | -0.21 ± 0.02 | -7.29 | 0.001 |
|  | Bag+Vaseline | 0.03 ± 0.03 | 0.87 | 0.431 |
| *Prunus lusitanica* | Bag | -0.59 ± 0.09 | -5.97 | 0.003 |
|  | Bag+Vaseline | -0.37 ± 0.03 | -10.68 | <0.001 |
| *Scrophularia glabrata* | Bag | -0.26 ± 0.07 | -3.37 | 0.027 |
|  | Bag+Vaseline | -0.12 ± 0.01 | -6.66 | 0.002 |

**Table S3.** Statistic results about the effect of the storage on the changes of ΔΨ_w_ or ΔRWC with time (two-way repeated measures ANOVA, experiment 1).

| **Species** | **Parameter** | **Storage** | | **Time** | | **Storage x Time** | |
| --- | --- | --- | --- | --- | --- | --- | --- |
|  |  | **F** | **P** | **F** | **P** | **F** | **P** |
| *Solanum nigrum* | ΔΨ_w_ | 52.23 | 0.002 | 247.50 | <0.001 | 34.72 | 0.004 |
|  | ΔRWC | 3.93 | 0.118 | 181.67 | <0.001 | 114.94 | <0.001 |
| *Morella faya* | ΔΨ_w_ | 35.56 | 0.004 | 13.63 | 0.021 | 34.04 | 0.004 |
|  | ΔRWC | 4.75 | 0.095 | 98.18 | <0.001 | 13.94 | 0.020 |
| *Prunus lusitanica* | ΔΨ_w_ | 3.78 | 0.124 | 87.52 | <0.001 | 4.07 | 0.114 |
|  | ΔRWC | 2.03 | 0.227 | 32.43 | 0.005 | 2.29 | 0.204 |
| *Scrophularia glabrata* | ΔΨ_w_ | 8.34 | 0.045 | 24.63 | 0.008 | 2.81 | 0.169 |
|  | ΔRWC | 0.04 | 0.846 | 165.49 | <0.001 | 8.41 | 0.044 |

**Table S4**. Evolution of water potential (Ψ_w_) and relative water content (RWC) at 25, 50 or 240 min after excision of leaves stored with the “vaseline plus humid bag” method. Note that ΔΨ_w_ was calculated comparing values from two different adjacent leaves collected simultaneously (Ψ_w,0_ and Ψ_w,x_, being x=20, 50 or 250 min), while ΔRWC compares values taken in the same leaf (RWC_0_ and RWC_x_). ID corresponds to the branch were leaves of both treatments were collected.

| **Parameter** | **ID** | ***Viburnum rigidum*** | | | | ***Solanum nigrum*** | | | | ***Prunus lusitanica*** | | | |
| --- | --- | --- | --- | --- | --- | --- | --- | --- | --- | --- | --- | --- | --- |
|  |  | **0** | **20** | **50** | **250** | **0** | **20** | **50** | **250** | **0** | **20** | **50** | **250** |
| Ψ_w_ | 1 | -0.39 | -0.40 | -0.40 | -0.46 | -0.65 | -0.64 | -0.68 | -0.79 | -0.49 | -0.51 | -0.51 | -0.79 |
|  | 2 | -0.48 | -0.55 | -0.55 | -0.6 | -0.67 | -0.56 | -0.78 | -0.77 | -0.55 | -0.60 | -0.58 | -0.67 |
|  | 3 | -0.39 | -0.47 | -0.45 | -0.64 | -0.57 | -0.62 | -0.59 | -0.76 | -0.48 | -0.50 | -0.58 | -0.62 |
|  | 4 | -0.46 | -0.45 | -0.47 | -0.54 | -0.82 | -0.71 | -0.91 | -0.93 | -0.49 | -0.55 | -0.52 | -0.66 |
|  | 5 | -0.51 | -0.51 | -0.48 | -0.52 | -0.61 | -0.63 | -0.70 | -0.82 | -0.47 | -0.45 | -0.42 | -0.58 |
|  | MEAN | **-0.44** | **-0.47** | **-0.47** | **-0.55** | **-0.66** | **-0.63** | **-0.73** | **-0.81** | **-0.49** | **-0.52** | **-0.52** | **-0.66** |
|  | SE | **0.02** | **0.02** | **0.02** | **0.03** | **0.04** | **0.02** | **0.04** | **0.03** | **0.01** | **0.02** | **0.02** | **0.03** |
| ΔΨ_w_ | 1 | - | -0.01 | -0.01 | -0.07 | - | 0.01 | -0.03 | -0.14 | - | -0.02 | -0.02 | -0.30 |
|  | 2 | - | -0.07 | -0.07 | -0.12 | - | 0.11 | -0.11 | -0.10 | - | -0.05 | -0.03 | -0.12 |
|  | 3 | - | -0.08 | -0.06 | -0.25 | - | -0.05 | -0.02 | -0.19 | - | -0.02 | -0.1 | -0.14 |
|  | 4 | - | 0.01 | -0.01 | -0.08 | - | -0.11 | -0.09 | -0.11 | - | -0.06 | -0.03 | -0.17 |
|  | 5 | - | 0.0 | 0.03 | -0.01 | - | -0.02 | -0.09 | -0.21 | - | 0.02 | 0.05 | -0.11 |
|  | MEAN | **-** | **-0.03** | **-0.02** | **-0.10** | **-** | **0.03** | **-0.06** | **-0.15** | **-** | **-0.02** | **-0.02** | **-0.16** |
|  | SE | **-** | **0.01** | **0.01** | **0.04** | **-** | **0.03** | **0.02** | **0.02** | **-** | **0.01** | **0.02** | **0.03** |
| RWC_0_ | 1 | 98.1 | 98.7 | 96.2 | 97.0 | 91.5 | 94.2 | 94.9 | 96.2 | 96.5 | 97.4 | 95.0 | 95.2 |
|  | 2 | 97.8 | 96.0 | 95.9 | 96.6 | 94.2 | 95.7 | 93.6 | 96.1 | 97.6 | 97.1 | 97.6 | 98.1 |
|  | 3 | 98.6 | 96.9 | 98.3 | 97.9 | 90.1 | 94.5 | 95.7 | 96.4 | 97.8 | 98.6 | 98.1 | 98.8 |
|  | 4 | 97.5 | 96.3 | 96.1 | 98.4 | 91.7 | 95.2 | 92.8 | 92.5 | 97.4 | 97.7 | 98.1 | 96.9 |
|  | 5 | 98.1 | 96.6 | 97.2 | 96.9 | 93.1 | 95.6 | 95.1 | 95.1 | 97.5 | 97.2 | 97.6 | 91.0 |
|  | MEAN | **98.0** | **96.9** | **96.7** | **97.3** | **92.1** | **95.0** | **94.4** | **95.2** | **97.3** | **97.6** | **97.2** | **96.0** |
|  | SE | **0.1** | **0.4** | **0.4** | **0.3** | **0.7** | **0.3** | **0.5** | **0.7** | **0.2** | **0.2** | **0.6** | **1.3** |
| RWC_x_ | 1 | - | 98.7 | 96.4 | 96.6 | - | 94.2 | 94.6 | 94.4 | - | 97.4 | 95.2 | 94.8 |
|  | 2 | - | 95.8 | 96.0 | 95.6 | - | 95.4 | 93.6 | 94.5 | - | 97.2 | 97.7 | 97.6 |
|  | 3 | - | 96.7 | 98.1 | 97.5 | - | 94.0 | 93.9 | 94.5 | - | 98.1 | 97.8 | 98.1 |
|  | 4 | - | 96.3 | 96.0 | 97.9 | - | 95.4 | 92.2 | 90.7 | - | 97.4 | 98.2 | 96.7 |
|  | 5 | - | 96.8 | 97.2 | 97.2 | - | 95.9 | 94.9 | 93.5 | - | 97.2 | 97.5 | 90.5 |
|  | MEAN | **-** | **96.8** | **96.7** | **96.9** | **-** | **94.9** | **93.8** | **93.5** | **-** | **97.4** | **97.3** | **95.5** |
|  | SE | **-** | **0.5** | **0.4** | **0.4** | **-** | **0.3** | **0.4** | **0.7** | **-** | **0.1** | **0.5** | **1.3** |
| ΔRWC | 1 | - | 0.0 | 0.2 | -0.4 | - | 0.0 | -0.3 | -1.8 | - | 0.0 | 0.2 | -0.4 |
|  | 2 | - | -0.2 | 0.1 | -1 | - | -0.3 | 0.0 | -1.6 | - | 0.1 | 0.1 | -0.5 |
|  | 3 | - | -0.2 | -0.2 | -0.4 | - | -0.5 | -1.8 | -1.9 | - | -0.5 | -0.3 | -0.7 |
|  | 4 | - | 0.0 | -0.1 | -0.5 | - | 0.2 | -0.6 | -1.8 | - | -0.3 | 0.1 | -0.2 |
|  | 5 | - | 0.2 | 0.0 | 0.3 | - | 0.3 | -0.2 | -1.6 | - | 0.0 | -0.1 | -0.5 |
|  | MEAN | **-** | **-0.0** | **0.0** | **-0.4** | **-** | **-0.0** | **-0.5** | **-1.7** | **-** | **-0.1** | **0.0** | **-0.4** |
|  | SE | **-** | **0.0** | **0.0** | **0.2** | **-** | **0.1** | **0.3** | **0.0** | **-** | **0.1** | **0.1** | **0.1** |
